# A multimodality imaging model to track viable breast cancer cells from single arrest to metastasis in the mouse brain

**DOI:** 10.1038/srep35889

**Published:** 2016-10-21

**Authors:** Katie M. Parkins, Amanda M. Hamilton, Ashley V. Makela, Yuanxin Chen, Paula J. Foster, John A. Ronald

**Affiliations:** 1 Robarts Research Institute, The University of Western Ontario, London, Ontario, Canada; 2 The Department of Medical Biophysics, The University of Western Ontario, London, Ontario, Canada; 3 Lawson Health Research Institute, London, Ontario, Canada

## Abstract

Cellular MRI involves sensitive visualization of iron-labeled cells *in vivo* but cannot differentiate between dead and viable cells. Bioluminescence imaging (BLI) measures cellular viability, and thus we explored combining these tools to provide a more holistic view of metastatic cancer cell fate in mice. Human breast carcinoma cells stably expressing Firefly luciferase were loaded with iron particles, injected into the left ventricle, and BLI and MRI were performed on days 0, 8, 21 and 28. The number of brain MR signal voids (i.e., iron-loaded cells) on day 0 significantly correlated with BLI signal. Both BLI and MRI signals decreased from day 0 to day 8, indicating a loss of viable cells rather than a loss of iron label. Total brain MR tumour volume on day 28 also correlated with BLI signal. Overall, BLI complemented our sensitive cellular MRI technologies well, allowing us for the first time to screen animals for successful injections, and, in addition to MR measures of cell arrest and tumor burden, provided longitudinal measures of cancer cell viability in individual animals. We predict this novel multimodality molecular imaging framework will be useful for evaluating the efficacy of emerging anti-cancer drugs at different stages of the metastatic cascade.

Breast cancer is the second most common cancer in both American and Canadian women^1^. The majority of breast cancer-associated mortality is due to metastasis; the dissemination of cancer cells from the primary tumour to other parts of the body, rather than the presence of a primary tumour. Therefore, the clinical need to better understand and prevent breast cancer metastasis is high.

A number of *in vivo* imaging modalities can be used to measure tumour size, location and metastatic burden such as positron emission tomography (PET), ultrasound (US), magnetic resonance imaging (MRI), computed tomography (CT), single photon emission computed tomography (SPECT) and optical imaging. Among these, MRI continues to be one of the most employed modalities for studying cancer due to its high resolution and soft tissue contrast without the use of ionizing radiation^2^.

Cellular MRI is an established tool to non-invasively visualize and track specific cell populations *in vivo*. This technique uses iron oxide nanoparticles to label cells in culture or *in situ*, enhancing their detectability by MRI^3^. The presence of intracellular iron causes a distortion in the magnetic field which leads to signal loss in iron-sensitive images^3^. Typically, millions of intracellular ultrasmall iron oxide particles (USPIOs) are needed to be detected by MRI, however Shapiro *et al*. were the first to show that single, micron-sized iron particles (MPIOs) can be loaded into cells and allow for single cell detection^4^. Wu *et al*. showed that these particles can also be used to label *in situ* for successful tracking of immune cells^5^. Although the amount of iron within these particles is significantly more than in the USPIOs, many cellular MRI studies have found that there is minimal impact on cell function or phenotype^6^,^7^,^8^. While we and others have used cellular MRI extensively in various scenarios, this technology has its limitations. First, is its limited ability to definitively differentiate between dead and viable cells. Furthermore, when a cell dies the iron label may be transferred to phagocytic bystander cells leading to false positive imaging results^9^. This was demonstrated by Winter *et al*. who injected both dead and living iron-labeled human epicardium-derived cells (EPDCs) into the infarcted heart of immune compromised mice^10^. The aim was to distinguish viable from dead cells by following the hypointensity of signal voids over time. They found that a difference could not be seen between signal intensity, void number, size or localization of live cells that migrate to the infarcted area, and dead cells that are taken up by macrophages or other bystander cells^10^. Thus a complementary imaging tool to cellular MRI that could provide direct measures of cellular viability over time would enable a more complete picture of cell fate in preclinical models.

Similar to cellular MRI technology, bioluminescence imaging (BLI) with the reporter firefly luciferase (FLuc) has been adapted by many researchers as a valuable cell tracking tool in preclinical models of cancer metastasis. The intensity and location of the bioluminescent signal can provide insights into the biology of neoplastic cells, with respect to their biodistribution and proliferative potential. FLuc BLI requires adenosine triphosphate (ATP) as a cofactor and so the BLI signal is directly proportional to the number of viable cells at a particular location^11^. This has been shown to provide corresponding information to tumour volume measurements with more structure-based imaging such as CT or MRI. This can be crucial in models of treatment response where the size or morphology of a tumour may not change but the amount of viable tissue within the tumour will be altered^12^. Similarly, when a tumour naturally becomes necrotic as it becomes larger, volume measurements can overestimate the number of viable cancer cells^12^. Furthermore, BLI is a highly sensitive technology that can enable the visualization and quantification of cancer cells at a stage where tumours are not yet detectable by these other relatively less affordable and less sensitive imaging modalities^13^. Given its high sensitivity and ability to provide measures of cellular viability, in this study we evaluated whether BLI can be used in conjunction with cellular MRI to follow cancer cell fate from their initial arrest in the brain to the formation of overt tumors in a well-established mouse model of breast cancer metastasis. We demonstrate that BLI complements well with our sensitive cellular MRI technologies, allowing us for the first time to get direct longitudinal measures of whole-brain single cell arrest, tumour volumes, and cancer cell viability, providing a more holistic view of transplanted cancer cell fate in living subjects. Importantly, combining of these imaging technologies should be broadly applicable to numerous preclinical models of experimental metastasis.

## Materials and Methods

### Cell Labelling and Transduction Procedure

Brain seeking human breast carcinoma cells (JIMT1-BR3) were engineered to stably co-express FLuc and GFP following transduction with an LVP020 lentiviral vector (GenTarget Inc., CA, USA). These cells were transduced and generously gifted by Dr. Patricia Steeg’s lab. Cells were maintained in DMEM containing 10% FBS at 37 °C and 5% CO^2^. For iron labeling, 2 × 10^6^ cells were plated in a 75 cm^3^ flask, supplemented with DMEM containing 10% FBS, and allowed to adhere for 24 hours. Then cells were incubated for an additional 24 hours with 10 mL media containing 25μg/mL of MPIO beads (0.9 um in diameter, 63% magnetite, labeled with Flash Red; Bangs Laboratory, Fishers, IN, USA). Cells were washed once in the flask with Hanks balanced salt solution (HBSS) and then trypsinized with 0.25% Trypsin-EDTA. The cells were then collected and thoroughly washed three more times with HBSS to remove unincorporated MPIO before cell injection and *in vitro* evaluation.

### *In Vitro* Studies

To evaluate the relationship between cell number and BLI signal, cells were seeded in 24-well plates in 0.5 mL of growth medium at concentrations of 5 × 10^3^, 1 × 10^4^, 2 × 10^4^ and 5 × 10^4^ cells per well. Cells were allowed to adhere for 24 hours and then 5μL of D-luciferin (30 mg/mL; Perkin Elmer) was added to the growth medium 5 minutes prior to BLI using a hybrid optical/Xray scanner (*In Vivo* FX PRO; Bruker formerly Kodak).

To evaluate if MPIO labeling effects BLI signal, cells were seeded in 24-well plates in 2 mL of growth medium with 1 × 10^4^ cells per well. Cells were allowed to adhere for 24 hr and then 25μg/mL of MPIO was added to the growth medium for half of the wells. Cells were then incubated for an additional 24 hr before 5μL of D-luciferin (30 mg/mL) was added for BLI.

### Animal Model

The animals were cared for in accordance with the standards of the Canadian Council on Animal Care, and under an approved protocol of the University of Western Ontario’s Council on Animal Care. To deliver MPIO-labeled FLuc/GFP + cells into the brain, 1.75 × 10^5^ cells were injected into the left ventricle of 12 female nu/nu mice (6–7 weeks old; Charles River Laboratories, Wilmington, MA, USA). Cells were suspended in 0.1 mL of HBSS and image-guided slow injections into the left ventricle were performed using a Vevo 2100 ultrasound system (VisualSonics Inc.).

### Animal Work Design

**Figure 1:**
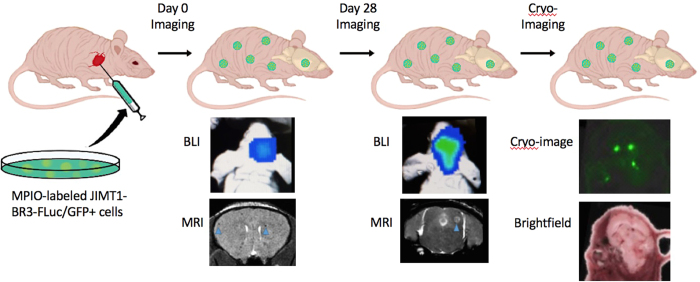
Illustration of MRI, BLI and fluorescence cryo-imaging of metastases in mice with intracardiac injection of human breast carcinoma cells. Original image courtesy of Chelsey Gareau.

Figure 1 illustrates the 4-week timeline of our *in vivo* multimodality imaging model. Twelve mice received intracardiac injections of 1.75 × 10^5^ MPIO-labeled JIMT1-BR3-FLuc/GFP + cells on day 0. BLI signal was detectable one-hour post injection and was used to differentiate between mice that had a successful intracardiac injection and mice that did not. Mice that did not have successful injections were excluded from the remainder of the study. Mice that had successful injections moved onto day 0 MRI four hours post injection. These mice were then imaged with both BLI and MRI on days 8, 21 and 28. After endpoint imaging, one mouse was sacrificed for cryofluorescence imaging and the rest were sacrificed for histology.

### BLI Procedure

All *in vivo* BLI was performed on a hybrid optical/Xray scanner (*In Vivo* FX PRO; Bruker formerly Kodak). Mice were anesthetized with isofluorane (2% in 100% oxygen) using a nose cone attached to an activated carbon charcoal filter for passive scavenging. Approximately one hour following cell injection whole body BLI imaging was used to screen mice for successful intracardiac injection on day 0. Only mice with BLI signal from the brain proceeded to MRI (i.e., only mice with successful intracardiac injections). On days 0, 8, 21 and 28, mice received 150 μL of D-luciferin (30 mg/mL) intraperitoneally and BLI images were captured every 5 minutes for up to 35 minutes.

### MRI Procedure

All MRI scans were performed on a 3T GE clinical MR scanner (General Electric) using a custom-built gradient coil and a custom-built solenoidal mouse brain radiofrequency coil^3^,^14^. Mice were anesthetized with isofluorane (2% in 100% oxygen) using a nose cone attached to an activated carbon charcoal filter for passive scavenging and images were obtained using a 3D balanced steady state free precession (bSSFP) imaging sequence [Fast Imaging Employing Steady State Acquisition (FIESTA) on the GE system] which has been previously optimized for iron detection^15^. Mice were imaged on days 0, 8, 21 and 28. The scan parameters for days 0 and 8 were: repetition time (TR) = 8 ms, echo time (TE) = 4 ms, bandwidth (BW) = 41.7 kHz, flip angle (FA) = 35 degrees, averages (NEX) = 2, phase cycles = 4, matrix = 150 × 150. Total scan time was approximately 15 minutes per mouse. For days 21 and 28, a longer scan time was required for tumour detection and so imaging parameters were: TR = 10 ms, TE = 5 ms, BW = 12.5 kHz, FA = 35 degrees, NEX = 2, phase cycles = 8, matrix = 150 × 150. Total scan time was approximately 35 minutes per mouse.

### Image Analysis

Brain BLI signal was measured using region-of-interest (ROI) analysis using the Bruker Molecular Imaging Software. An ROI was drawn around the brain, the mean photon flux (photons/second/mm^2^) was measured, and the peak value over the 30-minute imaging session was used for each mouse at each time point. MRI images were analyzed using OsiriX software (Pixmeo, SARL, Bernex, Switzerland). The number of dark pixels within the total brain volume was also determined from day 0 images; The brain was outlined as a region of interest where a threshold value is set based on the mean value of signal void ±2 standard deviations. The total number of black pixels under this threshold value was obtained from the entire tumour volume signal intensity histogram. For days 0 and 8 images, signal voids were also manually counted in every 8^th^ slice. The sum of all slices was then multiplied by 4 to account for a standard signal void that goes through an average of two slices. For days 21 and 28, brain metastases were manually traced by a single observer. 3D tumor volumes were reconstructed using the OsiriX volume algorithm from the manual segmentation of a region of interest around each tumor boundary in every bSSFP image slice for each mouse.

### Histology

At endpoint, mice were sacrificed by pentobarbital overdose and perfusion fixed with 4% paraformaldehyde. Mouse brains were removed and cryopreserved in ascending concentrations of sucrose (10, 20, and 30% w/v) in distilled water for 1 hour each. Brains were immersed in optimal cutting temperature (OCT) compound, oriented in a sectioning plane parallel to that of MRI, and frozen using liquid nitrogen. Contiguous 10-μm frozen sections were collected and stained using the following: hematoxylin and eosin (H&E) to visualize tumour morphology, firefly luciferase antibody (Abcam Inc., Cambridge, MA,USA; product # ab21176, dilution factor 1:1000) to identify luciferase positive cells, and Perl’s Prussian blue (PPB) staining to visualize iron. Stained sections were imaged using a Zeiss 510 laser scanning confocal microscope and GFP-positive cancer cells were also imaged.

### Cryo-Fluorescence Imaging

After endpoint MR imaging (day 28), one mouse was sacrificed by pentobarbital overdose and then flash frozen in OCT freezing medium by liquid nitrogen immersion. The entire mouse was sectioned and optically imaged every 50 μm using a CryoViz^TM^ (Bioinvision Inc., Cleveland, OH) cryo-imaging device. Block-face images were collected with an in plane resolution of 10.5 × 10.5 μm^2^. Brightfield and fluorescent images were acquired, stitched together and visualized using proprietary software (Bioinvision Inc).

### Statistics

We evaluated the effect of MPIO labeling on BLI signal in the JIMT1-BR3/FLuc-GFP cell line using a two tailed t-test. Pearson correlational analysis of cell number to photon flux in culture was performed as well as on *in vivo* BLI and MRI data on days 0 and 28. For all tests, a nominal p-value < 0.05 was considered statistically significant.

## Results

### *In Vitro* Studies

**Figure 2:**
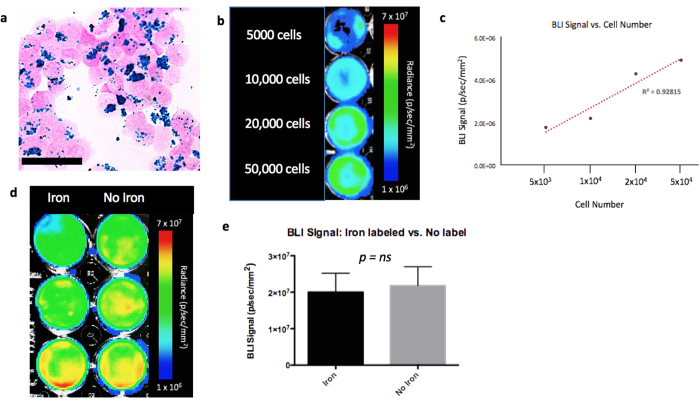
*In Vitro* Experiments (**a**) Perl’s Prussian blue stain identifies iron labelled cells in blue (scale bar x 100). (**b**) JIMT1BR3-Fluc/GFP + cells seeded at various concentrations (**c**) A strong linear correlation is seen between cell number and BLI signal; R^2^ = 0.928 (**d**) MPIO labeled JIMT1BR3-Fluc/GFP + cells (L) and non labeled JIMT1BR3-Fluc/GFP + cells (R). (**e**) There was no significant difference in BLI signal detected in cells that were labeled with MPIO and cells that were not labeled (ns, p value = 0.82). These results are representative of three experiments with three wells of each condition.

Figure 2a shows the JIMT1-BR3/FLuc-GFP cell line was efficiently labelled with MPIO. The Perl’s Prussian blue stain shows intracellular iron in blue within the breast cancer cells that appear pink. JIMT1-BR3/FLuc-GFP cells were seeded at 5 × 10^3^, 1 × 10^4^, 2 × 10^4^, and 5 × 10^4^ cells per well and BLI was performed. A significant positive correlation was seen between the number of JIMT1-BR3/FLuc-GFP cells seeded per well and BLI signal (R^2^ = 0.928). More specifically, as cell number increased, BLI signal also increased (Fig. 2b,c). There was no significant difference in BLI signal detected in cells that were labeled with MPIO (*M* = 2.00 × 10^7^ ± 5.17 × 10^6 ^p/s/mm^2^) and cells that were not labeled (*M* = 2.18 × 10^7^ ± 5.21 × 10^6 ^p/s/mm^2^) (*ns, p value* = 0.82; Fig. 2d,e). This suggests that MPIO labelling has no significant quenching effect on BLI signal. These results are from three independent experiments with three replicates of each condition.

### *In Vivo* Studies

**Figure 3:**
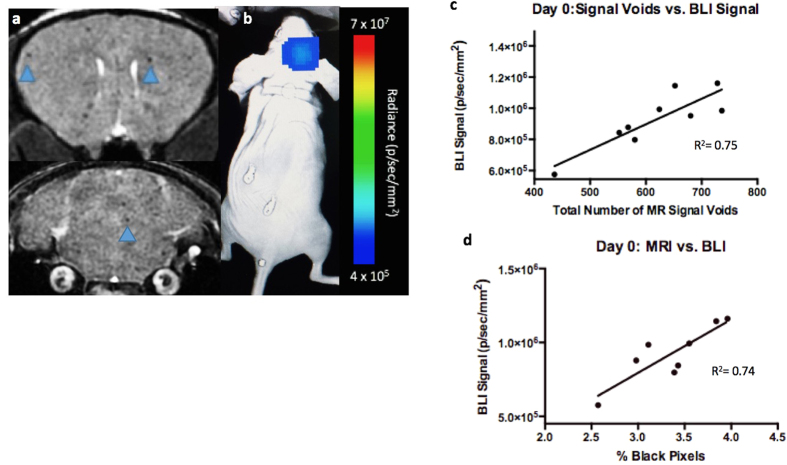
Day 0 Imaging; (**a**) single arrested cells could be visualized as signal voids (blue arrows) in day 0 MR images. (**b**) BLI signal was detectable in the brain on day 0. (**c**) A significant correlation was found between total number of signal voids measured from day 0 MRI scans and day 0 BLI signal in the brain (R^2^ = 0.75, *p* < 0.01). (**d**) A significant correlation was found between total percentage of black pixels measured from day 0 MRI scans and day 0 BLI signal in the brain (R^2^ = 0.74, *p* < 0.01).

Figure 3 shows image data from day 0. On day 0, iron labeled cells were visualized as signal voids by MRI, distributed throughout the brain (Fig. 3a). On average, 609± 98 discrete, signal voids per brain were quantified throughout the brains in MRI images from the nine animals that had successful injections of 1.75 × 10^5^ MPIO-labeled cells. As mentioned, BLI signal was also detected in the brain of these nine mice on day 0 (Fig. 3b). Three of twelve mice did not have a successful intracardiac injection and showed signal in other organs such as the lungs or abdominal cavity but not in the brain. These mice were excluded from the remainder of the study. Figure 3c shows that the number of signal voids measured on day 0 by MRI showed a significant correlation with BLI signal in the brain on day 0 (R^2^ = 0.75, *p* < 0.01). Percent black pixels were also measured from day 0 MRI images with the average percent black pixels within the whole brain being 3.35%; and showed a significant correlation with day 0 BLI signal in the brain (R^2^ = 0.74, *p* < 0.01) (Fig. 3d).

**Figure 4:**
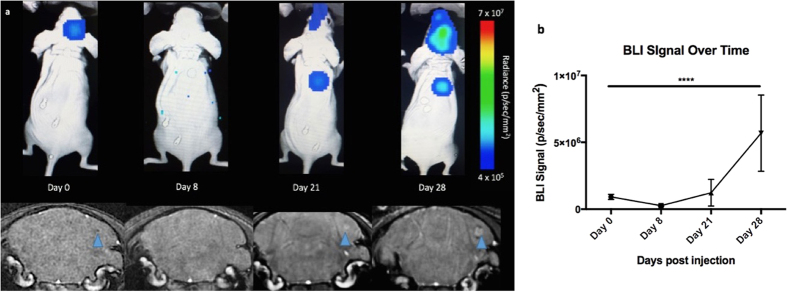
MRI and BLI over time (**a**) Representative BLI (top) and MRI images (bottom) of one mouse imaged on days 0, 8, 21 and 28; blue arrow shows signal void progressing into tumour (**b**) BLI signal in the brain over time. This graph is representative of 8 mice that made it to endpoint. A one-way ANOVA was performed and all time points were found to be significantly different (*p* < 0.0001).

Figure 4a shows imaging data over time and illustrates the MRI and BLI signal loss and recurrence over time with tumour development. Notably, on day 8, there were few to no voids seen by MRI and no detectable BLI signal in the brain; this is because the majority of cancer cells die and are cleared and those that remain are below our current BLI detection threshold. On day 21, three of nine mice showed BLI signal in the brain as well as small tumours detected by MRI. The remaining six mice did not have detectable tumours with either MRI or BLI at day 21. On day 28, these three mice had increased BLI signal and larger brain metastases detected by MRI.

When we graph BLI signal over time, we found that the signal dropped at day 8 when the cells were cleared but returned again when tumours started to form by day 21 (Fig. 4b). A one-way ANOVA was performed and BLI signal in the brain was found to be significantly different at all time points (*p* < 0.0001).

**Figure 5:**
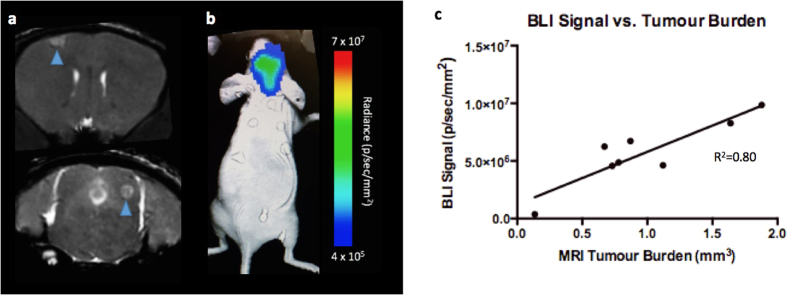
Day 28 Imaging (**a**) Brain metastases (blue arrows) appear as region of hyperintensity in day 28 MR images. (**b**) Tumours were also detected using BLI on day 28. (**c**) A significant correlation was found between total brain tumour burden measured by MRI at endpoint (day 28) and BLI signal in the brain (R^2^ = 0.80, *p* < 0.01).

Figure 5 shows image data from day 28. One mouse had to be sacrificed after day 21 imaging and so day 28 data is representative of eight mice. By day 28, brain metastases appeared as regions of hyperintensity by MRI and BLI signal was detected in the brains of all eight mice that had made it to endpoint (Fig. 5a,b). In BLI images, six out of eight mice also showed metastases in areas other than the brain. Total brain tumour burden measured by MRI at endpoint (day 28) showed a significant correlation with BLI signal in the brain on day 28 (R^2^ = 0.80, *p* < 0.01) (Fig. 5c).

### Histology and Whole-mouse Cryo-fluorescence Imaging

**Figure 6:**
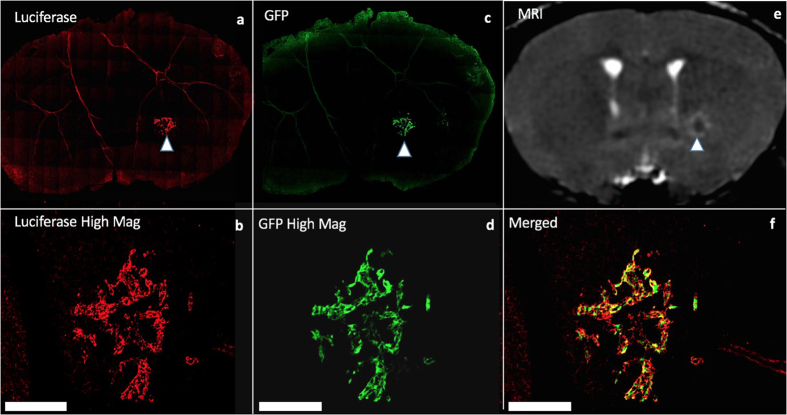
Fluorescence microscopy and immunohistochemistry (**a,b**) Firefly luciferase stain (**c,d**) green fluorescence protein (a/c low magnification, b/d high magnification) (**e**) Corresponding MRI image (**f**) Merged luciferase and GFP images at high magnification; blue arrows show tumour within whole slice image. Scale bars = 300 um.

**Figure 7:**
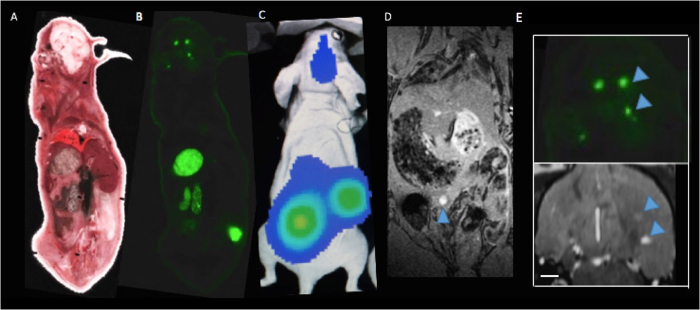
Cryoviz Imaging: (**A**) Brightfield image of cryoviz mouse imaged on day 28 (**B**) Fluorescence microscopy detects GFP + brain tumours and body tumours in same slice as brightfield image (**C**) Corresponding day 28 BLI and (**D**) Whole body MRI (**E**) Brain metastases (blue arrows) in cryo-image and corresponding MRI slice in the same orientation. Scale bar = 0.50 mm.

Mice were sacrificed 28 days after intracardiac cell injection. Dual fluorescence microscopy of immunostained sections demonstrated that the location of luciferase positive cells (labelled with red fluorescence) corresponded with the location of GFP expression (seen in green) (Fig. 6). These dual labelled cells also corresponded well with tumours seen in MR images suggesting that metastases are composed of the FLuc-GFP positive cells. In one mouse, the presence of tumours was also confirmed using cryo-fluorescence which has the ability to perform whole-body brightfield and fluorescence imaging throughout the entire mouse. Cryo-imaging allowed for the detection of metastases at day 28 in both the brain and other areas of the body (Fig. 7). Some of the metastases seen with cryo-imaging could be matched to MR tumours but not all of them; this is likely because some were too small to be detected with MRI. Whole body cryo-imaging also allowed for the localization of metastases seen with BLI. Due to light scattering and depth limitations, we cannot be certain where within the body the BLI signal is coming from; cryo-imaging allows us to further explore a region of signal and find out where exactly the tumour is located within the mouse body.

## Discussion

We have previously demonstrated that cellular MRI is a valuable cell tracking tool for preclinical investigation of brain metastatic breast cancer. However, it has limited ability to differentiate between dead and viable cells. Moreover, MRI can also provide measures of tumor volume in mouse models but this can potentially overestimate the number of viable cancer cells in those tumors. In this study we have for the first time combined cellular MRI with BLI to provide longitudinal measures of cellular viability and better study the entire metastatic cascade, from initial arrest to tumor formation, in a well-established breast cancer brain metastases mouse model. BLI complemented our cellular MRI techniques well, demonstrating for the first time that MR measures of early cell arrest are indeed viable cancer cells, that the decrease in MR void number from day 0 to day 8 is due to loss of arrested viable cells rather than loss of iron label, and that MR measures of brain tumor volumes at day 28 correlate well with BLI measures of cellular viability, indicating that tumors are mostly composed of viable cells in this model.

Past studies have found that the left cardiac ventricular delivery of cells is an ideal model for monitoring *in vivo* single cell detection; this is due to the fact that the delivery to a given organ is initially proportional to the percentage of cardiac output (%CO) to that organ. In this case, the %CO to the brain is ~9.5% and therefore for an injection of 175,000 cells, we can expect ~16,625 cells to be delivered to the brain microcirculation^16^. However, <1% of these cells are expected to be retained in the brain by 2 hours post injection^16^. On average, 609 ± 98 discrete, signal voids were visualized throughout the brain in day 0 MRI images. The MPIO used to label the JIMT1BR3-FLuc/GFP cells affects the MR signal by creating a blooming effect. As a result, a signal void appears much larger than the cell itself; making single cell detection feasible. It has been shown that a signal void is usually representative of a single cell, however it can also be representative of two or three cells clumped together^3^. Thus, the 400–700 cells we are detecting at day 0 may be closer to 1000 cells.

When compared to other imaging technologies, BLI is often characterized as relatively inexpensive, user friendly and highly sensitive. As a result, BLI can be used as a high-throughput screening tool for cell tracking studies. In this study we used BLI to screen for successful intracardiac injections on day 0. Mice that did not have a successful injection, did not have any bioluminescent signal detected in the brain and thus were excluded from the study. Prior to developing this model, each mouse would need to undergo a day 0 MRI scan to determine if the injection was successful by detecting arrested cells (i.e., signal voids). BLI can also be used to guide experimental design as well as predict or evaluate a region of interest in less developed animal models. In this study, whole body BLI allowed for the detection of metastases at sites that we had not previously detected or evaluated. Day 28 BLI images helped to predict suspected organs of metastasis for *ex vivo* analysis. To achieve sufficient resolution to detect iron-labelled cells in the brain, within a suitable scan time, only the brain is imaged with MRI and metastases in other parts of the body may have been overlooked if they were not prominent.

While MRI and BLI have been used separately as measures of overall tumour burden, primarily in models with singular tumours, the concurrent application of BLI and MRI to studying the entire process of brain metastasis has not been performed. In this work, BLI allowed for repetitive, non-invasive, whole body imaging and MRI based measurements were used to validate BLI measurements of single cell arrest and tumour burden in the mouse brain. There was a significant linear relationship between tumour volume measured by MRI and light output measured by BLI. Although our MRI based measurements may be a result of both dead and live tumour cells, phagocytic bystander cells (for iron measurements), and other tumour stromal cells, BLI signal is representative of viable tumour cells only. The correspondence between modalities at day 28 suggests that our metastases are made up of mostly viable cancer cells at this time point. If we had imaged at a later time point the correlation between modalities may become weaker as tumours progress and develop a necrotic core. Similarly, if treatment was given, a decrease in viable tumour tissue will affect the correlation between our multimodality tumour burden measurements. This is an important advantage of BLI when evaluating potential treatment paradigms, as a tumour may not look anatomically different in early stages of treatment but the amount of viable tissue within that tumour may change.

Cryo-imaging allowed for the detection of fluorescent micrometastases in the brain as well as other places in the body. While food in the abdominal cavity creates some autofluorescence this may be alleviated in the future using alfalfa-free diets. However, tumours detected with cryo-fluorescence imaging that appear to be in the middle right abdominal cavity and above the left hindlimb matched well with BLI images and appear much brighter than the autofluorescence from the food. Like MRI, the cryo-imaging can also allow us to localize and count the number of micrometastases in the brain where as the BLI image gives us one individual measure for all combined tumours. Some brain metastases found in MR images matched our cryo-fluorescence images well while others appeared too small to be detectable with MRI.

This study demonstrates how a multimodality imaging model that uses both MRI and BLI can overcome the limitations of using each modality independently. Although BLI can produce a measure of tumour burden within the brain, we are unable to collect information on the number, size or distribution of tumours within the region of interest. This can be a limiting factor in cancer metastasis models that use BLI only; numerous tumours or arrested cells that are close in proximity appear as one large region of signal due to light scattering, and at equivalent sizes tumours that are more superficial will appear to be brighter than those that are located deeper. In contrast, MRI can detect individual iron-loaded cells and provide information on tumour 3D location and size. These modalities also complement each other at different time points throughout the experiment. Our current BLI protocol was not sensitive enough to detect signal in the brain on day 8, even though we know there are in fact viable cancer cells there (i.e., tumours formed in the brain and signal voids were seen on day 8). Future work using newer luciferase substrates or BLI machines with more sensitive CCD cameras could overcome this limitation^17^,^18^. However, our cellular MRI technology was able to detect the limited number of cells that persisted in the brain on day 8. By day 21 tumours were easily detected by BLI in three of nine mice however, these tumours were very small and thus difficult to find with MRI. Tumours with minimal burden and MR contrast on day 21 may have been overlooked if BLI signal had not predicted metastases prior to MRI imaging.

## Conclusions

In this work we applied multimodality imaging to monitor the growth of metastatic breast cancer cells in the brain from single arrested cells to overt tumours. BLI complemented our sensitive iron-based cellular MRI technologies well, allowing us for the first time to get direct longitudinal measures of whole-brain single cell arrest, tumour burden, and cancer cell viability in the brain. Future work will extend these tools for whole-mouse MRI/BLI of metastatic burden, which will be extremely valuable for enabling an improved understanding of the fate of single cancer cells throughout the body and evaluation of current and emerging treatment paradigms.

## Additional Information

**How to cite this article**: Parkins, K. M. *et al*. A multimodality imaging model to track viable breast cancer cells from single arrest to metastasis in the mouse brain. *Sci. Rep.* **6**, 35889; doi: 10.1038/srep35889 (2016).

## Supplementary Material

Supplementary Figure Legend for Video

Supplementary Video
