# Supplementary material for: A multimodality imaging model to track viable breast cancer cells from single arrest to metastasis in the mouse brain
Source: Sci Rep. 2016 Oct 21;6:35889. doi: 10.1038/srep35889 (PMC5073295; doi:10.1038/srep35889)
Supplement: Supplementary Figure Legend for Video [file srep35889-s1.pdf]

A multimodality imaging model to track viable breast cancer cells from single arrest to metastasis in the mouse brain

Katie M. Parkins*[1,2], Amanda M. Hamilton[1], Ashley V. Makela[1,2], Yuanxin Chen[1]
Paula J. Foster[1,2], John A. Ronald[1,2,3]

[1]Robarts Research Institute, The University of Western Ontario, London, Ontario, Canada
[2]The Department of Medical Biophysics, The University of Western Ontario, London, Ontario, Canada
[3]Lawson Health Research Institute, London, Ontario, Canada

Corresponding Author: jronald@robarts.ca

Supplementary Information File

Supplementary Figure Legend for VIDEO file

Video of cryo-fluorescence imaging
